# Angiogenesis and multiple myeloma: Exploring prognostic potential of adrenomedullin

**DOI:** 10.1002/cam4.70250

**Published:** 2024-09-24

**Authors:** Angelos Giannakoulas, Panagiotis Stoikos, Evangelia Kouvata, Katerina M. Kontouli, Georgios Fotiadis, Georgia Stefani, Grigorios D. Amoutzias, George Vassilopoulos, Nikolaos Giannakoulas

**Affiliations:** ^1^ Laboratory of Hematology Department, Faculty of Medicine University of Thessaly Larissa Greece; ^2^ Hematology Department University Hospital of Larissa Larissa Greece; ^3^ Laboratory of Hygiene and Epidemiology, Faculty of Medicine University of Thessaly Larissa Greece; ^4^ Bioinformatics Laboratory, Department of Biochemistry & Biotechnology School of Health Sciences, University of Thessaly Larissa Greece

**Keywords:** adrenomedullin, angiogenesis, multiple myeloma, prognostication

## Abstract

**Background:**

Adrenomedullin (AM) is a multifunctional peptide which under basal conditions mainly regulates vasodilation and maintains vascular integrity but is also implicated in the pathogenesis of several malignancies, including multiple myeloma (MM). It has been shown that adrenomedullin is expressed by human myeloma cell lines and that it enhances MM‐driven angiogenesis. However, the clinical impact of AM remains unknown.

**Materials and Methods:**

On that basis, we enrolled 32 newly diagnosed multiple myeloma patients (NDMM) and studied the potential of AM as a prognostic biomarker.

**Results:**

We report that elevated levels of AM trend with suboptimal treatment response and inferior survival of NDMM patients.

Increased angiogenesis is a hallmark feature of multiple myeloma (MM) resulting in poor clinical outcomes of MM patients.^1^ Current knowledge suggests that tumor‐stroma interactions result in an imbalance between pro‐angiogenic and anti‐angiogenic modulators, which favors vascular development and tumor growth. Several molecules have been proposed to promote MM's angiogenic switch including VEGF, IL‐6/8, OPN, MMP1, and Adrenomedullin (AM).^2^, ^3^ AM is a multifunctional peptide which under basal conditions mainly regulates vasodilation and maintains vascular integrity.^4^ Yet, since the initial report by Kitamura et al.,^5^ a number of studies have linked adrenomedullin with the growth of several solid tumors, including prostate, lung, and breast cancers.^6^ We have previously shown that ADM gene is overexpressed in CD138+ cells from NDMM patients compared to healthy donors.^7^ It has also been shown that adrenomedullin is expressed in a hypoxia‐dependent and hypoxia‐independent manner by L363, LME‐1 and RPMI8226 human myeloma cell lines and that it enhances MM‐driven angiogenesis mainly by stimulating endothelial cell proliferation and endothelial tube formation.^3^ However, the impact of AM in prognosis, treatment response and survival of MM patients remains unknown. Here, the clinical significance of adrenomedullin was investigated.

We enrolled 32 newly diagnosed multiple myeloma (NDMM) patients (male 19, median age 69, range 44–86) diagnosed and treated in the Department of Hematology of University Hospital of Larissa, Greece. The enrollment period was from March 2019 to July 2023. Twenty additional volunteers (male 13, median age 67, range 50–78), 10 healthy donors and 10 patients with Hodgkin/non‐Hodgkin lymphomas (with no marrow infiltration as assessed by bone marrow biopsies and PET/CT scans), were also enrolled and served as internal controls. Diagnosis of MM was based on International Myeloma Working Group consensus criteria.^8^ Patients received either VCD or VRD based regiments. Response was evaluated after five cycles of induction therapy. Patient's baseline characteristics are presented in Table 1. All participants provided informed consent prior study entry and the study was approved by the Institutional Review Board of the Hospital and carried out in accordance with the declaration of Helsinki. All samples were taken at the time of diagnosis, prior treatment initiation. Bone marrow aspirates were collected in EDTA‐containing tubes and processed within 2 h after sampling.

**TABLE 1:** Patient's baseline characteristics.

Age in years (median, range)	69, 44–86
Gender, *n* (%)	
Male	19 (59%)
Female	13 (41%)
MM type, *n* (%)	
IgG	16 (50%)
IgA	7 (21%)
IgM	1 (4%)
Light chain only	8 (25%)
Bone marrow plasma cell infiltration (median %, range)	45% (15%–90%)
ISS, *n* (%)	
I	6 (19%)
II	9 (28%)
III	17 (53%)
R2‐ISS, *n* (%)	
I	5 (15%)
II	7 (22%)
III	16 (50%)
IV	4 (13%)
Cytogenetic Risk, *n* (%)	
Standard	25 (78%)
High	7 (22%)
Lytic Bone Lesions, *n* (%)	
None	8 (25%)
1–3	7 (21%)
>3	17 (54%)
SREs at diagnosis, *n* (%)	10 (31%)
Fractures	8 (25%)
Bone related RT or Surgery	8 (25%)
Presence of soft tissue plasmacytoma at diagnosis, *n* (%)	11 (34%)
Frontline treatment, *n* (%)	
VCD	16 (50%)
VRD	9 (28%)
DARA‐VCD	3 (9%)
DARA‐RD	2 (6.5%)
VRD‐Autologous	2 (6.5%)

Bone marrow mononuclear cells (BMMNCs) were separated using density gradient separation with Ficoll Paque Plus (Sigma‐Aldrich, USA) (Methods S1). Cells were washed with DPBS (Biowest, France), lysed with RBC lysis buffer (Cell Signaling Technology, USA) when appropriate, aliquoted and stored as dry pellet at −80°C until further analysis. Total RNA was extracted from BMMNCs using E.Z.N.A. Total RNA kit I (Omega‐Biotek Inc., USA) following manufacturer's instructions. RNA concentration was quantified at 260 nm using a Nanodrop 2000 spectrophotometer. Total RNA was reverse transcribed into cDNA in a 20 μL reaction volume using QuantiTect Reverse Transcription kit (Qiagen, Germany), cDNA was diluted and stored at −20C. An in‐house PCR assay amplifying exon 3–4 boundaries of GAPDH gene with primers flanking short intron 3–4 region was used to validate appropriate reverse transcription and elimination of any genomic DNA trace (Methods S2 and Figure S1). RT q‐PCR was carried out in a 36 well Rotor Gene Q (Qiagen) using CYBR green 1 as fluorescence dye. ACTB was used as housekeeping gene. Primers, targeting 3‐UTR regions, were used and their sequences were as follows: for ADM F: TTGTCCTCCCCTATTTTAAGACG, R: CTTCCACACAGGAGGTAATCAGTC and for ACTB F: TTTTTGTCCCCCAACTTGA, R: TGGCTGCCTCCACCCA. The cycling conditions included an initial incubation step at 95°C for 2 min followed by 40 cycles of 95°C for 15 s and 58°C for 40 s. Specificity of each reaction was confirmed with melt curve analysis (Figure 1A,B) and by resolving amplification products in a 2% agarose gel, stained with ethidium bromide (Figures S2, S3). RT q‐PCR reactions were conducted in duplicates and Ct values are presented as means. Gene expression levels were analyzed with the Livak method.^9^ Statistical analysis was conducted with R software (Methods S3).

**FIGURE 1:**
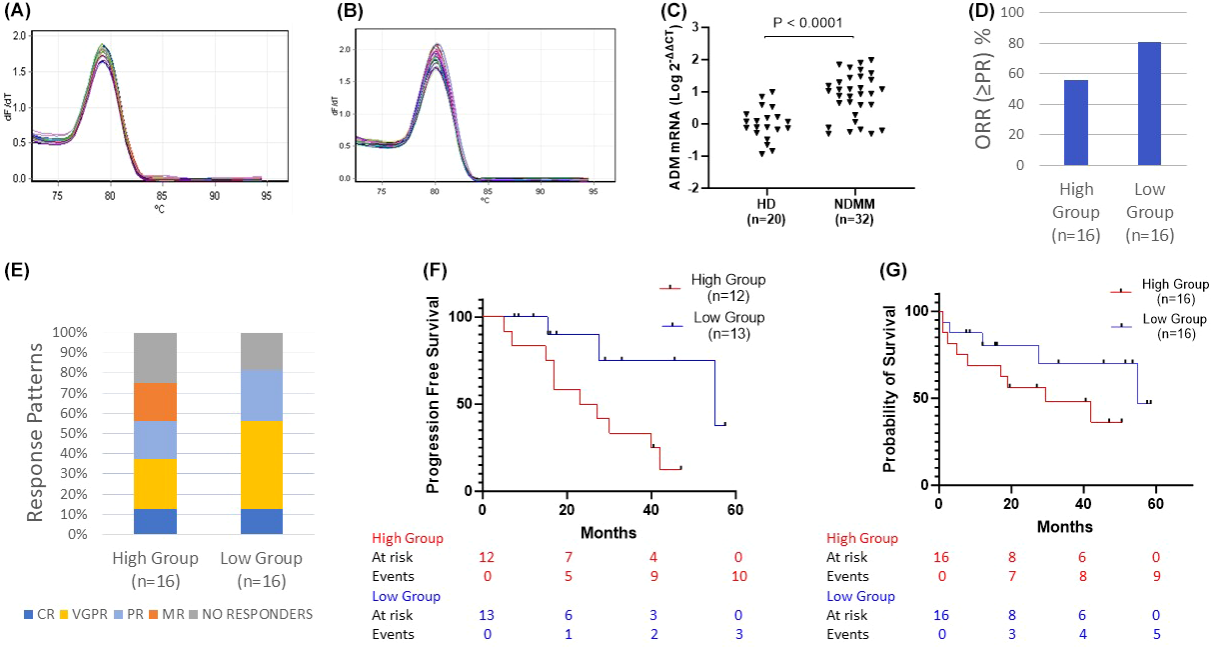
Exploring prognostic utility of Adrenomedullin in Multiple Myeloma. (A) Melt curve analysis of ACTB gene products. See the single peak at 78°C, indicating the specificity of qPCR reactions. (B) Melt curve analysis of ADM gene products. See the single peak at 80°C, indicating the specificity of qPCR reactions. (C) Relative gene expression analysis of AM between healthy donors (HD) and newly diagnosed multiple myeloma (NDMM) patients with the 2−^ΔΔCT^ Livak method. The y‐axis is on a log10 scale. (D). Overall response rates (ORR) (expressed as ≥ partial response) from NDMM patients with elevated AM expression measures (high group) and patients with lower measures (low group). Response was evaluated after 5 cycles of induction therapy. (E) Comparison of response depths and patterns between the 2 patient subgroups. (F) Progression Free Survival curves for patients who achieved MR or better post frontline treatment, stratified based on their expression of ADM gene. (G) Kaplan–Meier curves for the survival of NDMM patients, stratified based on their expression of ADM gene.

To establish the differential expression of AM in MM, we first compared AM levels of the ADM gene between NDMM patients and healthy donors. AM mRNA abundance was 10‐fold higher in the NDMM group compared to the HD group (*p* < 0.0001) (Figure 1C). Next, we investigated the prognostic potential of AM. We categorized patients into two equally numbered groups based on the expression levels of AM. The median DCT value of the NDMM population served as cut‐off point. The first group (High) comprised of patients with elevated expression measures of AM (*n* = 16, median DCT 4.6, range 2.7–5.8) and the second one (Low) comprised of patients with lower measures (*n* = 16, median DCT 7.8, range 6.1–10.3). The 2 groups did not differ in age, sex, percentage of bone marrow infiltration, ISS stage, R2‐ISS stage (*p* = 0.6, 0.7, 0.8, 0.9, 0.6 respectively) and were equally treated either with VRD‐based or VCD‐based regiments (Table S1).

The Overall response rate (≥ PR) was 56% (*n* = 9/16) for the high group and 81% (*n* = 13/16) for the low group (Figure 1D). The response patterns and depths are shown in Figure 1E. After a median follow up period of 23 months (range 1–57 months), 14 (87%) and 8 (50%) patients from the high and low expression group, respectively, have relapsed or died (*p* = 0.1) with a median time to progression or death of 16 (range 1–42) and 16.5 (range 1–55) months, respectively. We, next, calculated progression free survival (PFS) for those patients who achieved MR or better post frontline treatment (*n* = 25/32). We specifically excluded patients with refractory disease and patients with early mortality (<3 months) to assess the effect of AM in disease progression. The median PFS, estimated with the Kaplan–Meier method, was 25 and 55 months for the high and low expression group, respectively (logrank HR = 3.8, 95% CI ratio 1.2–11.3, *p* = 0.02) (Figure 1F). Additionally, Kaplan–Meier curves were used to calculate probability of survival for the whole cohort. The median estimated overall survival for patients with elevated AM measures was 29.5 months compared to 55 months for patients with lower AM measures (logrank HR = 2.1, 95% CI of ratio 0.7–6.1, *p* = 0.1) (Figure 1G).

In univariate analysis, age (HR = 1.6, *p* = 0.02), LDH levels (HR = 1.01, *p* = 0.002), b2‐microglobulin levels (HR = 1.15, *p* = 0.03), R2‐ISS stage 3 (HR = 2.4, *p* = 0.4), R2‐ISS stage 4 (HR = 5.4, *p* = 0.1) and levels of AM (for every DCT reduction by one unit; HR = 1.2, *p* = 0.1) were factors affecting survival. In multivariate analysis, age (HR = 1.1, *p* = 0.001) and R2‐ISS stage 4 (HR = 21.8, *p* = 0.01) were independent factors predicting poor survival whereas elevated levels of AM (expressed as reduced DCT values) increased the risk of death (for every DCT reduction by one unit; HR = 1.1, *p* = 0.4). Pearson and Spearman correlation analyses did not show any correlation of AM DCT values with age (*R* = −0.1), LDH (*R* = −0.3), b2‐microglobulin (*R* = −0.1), ISS (*R* = −0.06) and *R* ^2^‐ISS (*R* = −0.03) implying that the observed differences between the 2 groups (high/low) are probably due to AM expression and not a result of other confounding factors.

In conclusion, to our knowledge this is the first exploratory study that evaluated the prognostic potential of adrenomedullin in NDMM patients. Although, statistical significance was not reached (possibly due to the small sample size), our findings indicate that elevated levels of adrenomedullin trend with suboptimal treatment response and inferior survival of NDMM patients. Future large‐scale studies are needed to fully uncover the prognostic utility of AM and to evaluate whether more conventional and widely applicable methods, like serum's measurements of AM protein, can be used as a screening tool to identify high risk patients.

## AUTHOR CONTRIBUTIONS

**Angelos Giannakoulas:** Formal analysis (lead); investigation (lead); methodology (lead); writing – original draft (lead). **Panagiotis Stoikos:** Data curation (equal); methodology (equal). **Evangelia Kouvata:** Data curation (equal); methodology (equal). **Katerina M. Kontouli:** Formal analysis (equal); methodology (equal). **Georgios Fotiadis:** Data curation (equal); methodology (equal). **Georgia Stefani:** Data curation (equal); methodology (equal). **Grigorios D. Amoutzias:** Formal analysis (lead); writing – review and editing (lead). **George Vassilopoulos:** Formal analysis (lead); writing – review and editing (lead). **Nikolaos Giannakoulas:** Conceptualization (lead); formal analysis (lead); project administration (lead); supervision (lead); writing – review and editing (lead).

## ETHICS STATEMENT

The study was approved by the Institutional Review Board of the University Hospital of Larissa and carried out in accordance with the declaration of Helsinki.

## Supporting information


**Data S1:** Supporting Information.

## Data Availability

All data can be provided from the correspondence author upon reasonable request.
