# Supplementary material for: Angiogenesis and multiple myeloma: Exploring prognostic potential of adrenomedullin
Source: Cancer Med. 2024 Sep 24;13(18):e70250. doi: 10.1002/cam4.70250 (PMC11420937; doi:10.1002/cam4.70250)
Supplement: Supplementary file 1 — Data S1: Supporting Information. [file CAM4-13-e70250-s001.docx]

**SUPPLEMENTAL DATA**

**SUPPLEMENTAL METHODS**

**1. ISOLATION OF BONE MARROW MONONUCLEAR CELLS (BMMNCs):**

Bone marrow aspirates were collected in EDTA-containing tubes and processed within 2 hours after sampling. Bone marrow was diluted 1:1 with DPBS (Biowest, France) and was filtered through a 100μm pore to remove cell clumps, clots and bone fragments. 4 ml of diluted cell suspension was carefully layered over 4 ml of Ficoll Paque Plus (Sigma-Aldrich, USA) and an immediate centrifugation step (400g at 20 ^o^C for 30 minutes without brake) followed. The upper plasma layer was aspirated and the interphase was drawn for washing steps and further analyses.

**2. VALIDATION OF APPROPRIATE REVERSE TRANSCRIPTION AND ELIMINATION OF gDNA**

Approximately 100ng of diluted cDNA from each sample were subjected to a GAPDH-based double-reaction PCR assay to ensure appropriate reverse transcription and elimination of the genomic DNA during the RNA extraction - RNA reverse transcription procedure. A pair of intron-spanning primers for GAPDH gene (F: ACGGATTTGGTCGTATTGGGC, R: TTGACGGTGCCATGGAATTTG) were used. The cycling conditions included an initial denaturation step at 94 °C for 2 minutes, followed by 25 cycles of denaturation at 94 °C for 15 seconds, annealing at 60 °C for 15 seconds and elongation at 72 °C for 30 seconds. The PCR assay would result in a single product (157bp) if the genomic DNA was appropriately digested. In contrast, contamination of the cDNA solution with genomic DNA would result in an additional second product (247bp). (supplemental figure 1)

**3. STATISTICAL ANALYSES**

Univariate and multivariate analyses were carried out by Cox regression and included the following variables: age (as a continuous variable), LDH level, b2-microglobulin level, R2-ISS and levels of AM. We applied a backward elimination process to the included variables, using a 5% significance level to stay in the model, to identify the final set of relevant factors. Kaplan-Meier curves were used to calculate probability of survival. Pearson correlation, also known as linear correlation, was used for the linear relationship between two continuous variables, and Spearman correlation was used for categorical data. All reported p-values are two-sided and the threshold of 0.05 was set as a cut-off of significance level. All statistical analyses were conducted with R software (version 4.3.2.).

**SUPPLEMENTAL TABLE 1:**

Frontline treatments of the 32 NDMM patients, divided into 2 groups based on their expression of the ADM gene.

|  | **High AM Group (n=16)** | **Low AM Group (n=16)** |
| --- | --- | --- |
| **VCD** | 8 | 8 |
| **VRD** | 4 | 5 |
| **DARA-VCD** | 1 | 2 |
| **DARA-RD** | 1 | 1 |
| **VRD plus autologous** | 2 | 0 |

**SUPPLEMENTAL FIGURE 1:**

2% gel electrophoresis of GAPDH PCR products. See the single bands at 157 bp, indicating the RNA-based origin of DNA in the 3 cDNA samples.

**
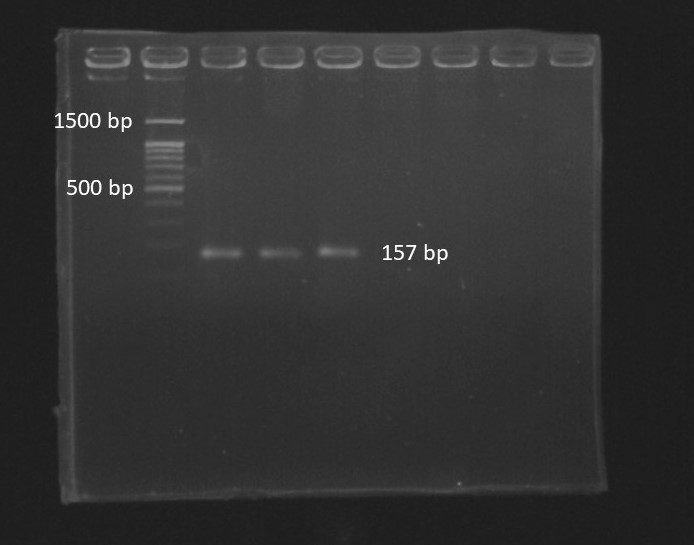
**

**SUPPLEMENTAL FIGURE 2:**

2% Gel electrophoresis of ADM gene qPCR products. See the single bands at 119 bp, indicating the specificity of reaction.

**
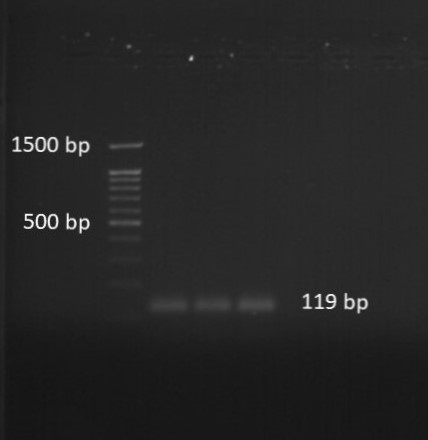
**

**SUPPLEMENTAL FIGURE 3: gel ACTB**

2% Gel electrophoresis of ACTB gene qPCR products. See the single bands at 66 bp.

**
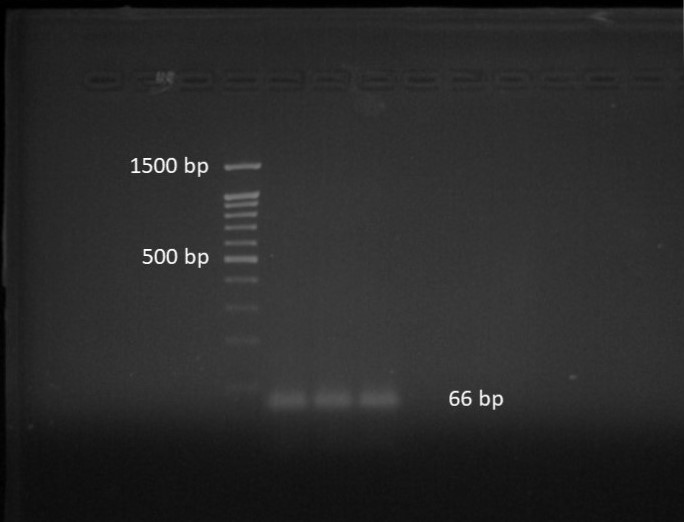
**
